# Serpins in cartilage and osteoarthritis: what do we know?

**DOI:** 10.1042/BST20201231

**Published:** 2021-04-12

**Authors:** David J. Wilkinson

**Affiliations:** Department of Musculoskeletal Biology and Ageing Sciences, Institute of Life Course and Medical Sciences, University of Liverpool, William Henry Duncan Building, 6 W Derby St, Liverpool L7 8TX, U.K.

**Keywords:** cartilage, metalloproteases, osteoarthritis, protease inhibitor, serine proteases, serpin

## Abstract

Serpins (serine proteinase inhibitors) are an ancient superfamily of structurally similar proteins, the majority of which use an elegant suicide inhibition mechanism to target serine proteinases. Despite likely evolving from a single common ancestor, the 36 human serpins have established roles regulating diverse biological processes, such as blood coagulation, embryonic development and extracellular matrix (ECM) turnover. Genetic mutations in serpin genes underpin a host of monogenic disorders — collectively termed the ‘serpinopathies’ — but serpin dysregulation has also been shown to drive pathological mechanisms in many common diseases. Osteoarthritis is a degenerative joint disorder, characterised by the progressive destruction of articular cartilage. This breakdown of the cartilage is driven by the metalloproteinases, and it has long been established that an imbalance of metalloproteinases to their inhibitors is of critical importance. More recently, a role for serine proteinases in cartilage destruction is emerging; including the activation of latent matrix metalloproteinases and cell-surface receptors, or direct proteolysis of the ECM. Serpins likely regulate these processes, as well as having roles beyond serine proteinase inhibition. Indeed, serpins are routinely observed to be highly modulated in osteoarthritic tissues and fluids by ‘omic analysis, but despite this, they are largely ignored. Confusing nomenclature and an underappreciation for the role of serine proteinases in osteoarthritis (OA) being the likely causes. In this narrative review, serpin structure, biochemistry and nomenclature are introduced, and for the first time, their putative importance in maintaining joint tissues — as well as their dysregulation in OA — are explored.

## Introduction

The serpin (serine proteinase inhibitor) superfamily of proteins is both ancient and unique. Found in all five kingdoms of life, they are likely to have evolved from a single common ancestor — termed ‘the archeserpin’ — approximately 500 million years ago [1,2]. Despite individual serpins being described for over a century, it was not until the early 1980s that structural similarities were observed between remarkably different proteins, when a unique protein family was uncovered [3]. The term ‘serpin’ was coined later, due to the observed antiproteinase activity [4] but it is now well understood that serpins can be both inhibitory or non-inhibitory.

### Serpin structure, mechanism, nomenclature and evolution

There are 36 protein-coding serpin family members in humans, of which 30 can inhibit proteinases [5]. Serpins are subdivided into ‘clades’ — A through to I — based on sequence similarity (Table 1). Outside of these groups, serpins exhibit surprisingly little sequence similarity, except for some conserved residues (e.g. Ser^53^ and Ser^56^) within the ‘shutter region’ in the core of the protein. This is an area of critical importance for the serpin mechanism, which is highlighted by mutations in these positions leading to several serious human serpinopathies; a collection of diseases resulting from serpin dysfunction [6]. However, despite only low sequence similarity, serpins are remarkably similar structurally; usually consisting of 7–9 α-helices surrounding 3 β-sheets and a large flexible region above the body of the protein, known as the reactive centre loop (RCL).

**Table 1.** The protein-coding serpins in humans

	Standardised nomenclature	Historical name(s)	RCL sequence (P4–P3–P2–P1⇣P1′–P2′–P3′–P4′)	Described proteinase target(s)
Clade A	SerpinA1	Alpha-1 antitrypsin; alpha-1 proteinase inhibitor	AIPM⇣SIPP	Neutrophil elastase; proteinase-3
	SerpinA2	Alpha-1 antitrypsin-related protein	EKAW⇣SKYQ	
	SerpinA3	Alpha-1 antichymotrypsin	ITLL⇣SALV	Cathepsin G
	SerpinA4	Kallistatin	IKFF⇣SAQT	Tissue kallikrein
	SerpinA5	Protein C inhibitor	FTFR⇣SARL	Activated protein C
	SerpinA6	Corticosteroid-binding globulin		Non-inhibitory
	SerpinA7	Thyroxin-binding protein		Non-inhibitory
	SerpinA8	Angiotensinogen		Non-inhibitory
	SerpinA9	Centerin	FIVR⇣SKDG	—
	SerpinA10	Protein-Z dependent proteinase inhibitor	ITAY⇣SMPP	Factor Xa; Factor XIa
	SerpinA11	—		
	SerpinA12	Vaspin	TLPM⇣ETPL	Kallikrein 7
Clade B	SerpinB1	Leukocyte elastase inhibitor (LEI); monocyte/neutrophil elastase inhibitor (MNEI)	ATFC⇣MLMP	Neutrophil elastase, cathepsin G, proteinase-3, cathepsin L
	SerpinB2	Plasminogen-activator inhibitor (PAI-2)	MTGR⇣TGHG	uPA
	SerpinB3	Squamous cell carcinoma antigen 1 (SCCA1)	GFGS⇣SPTS	Cathepsin K, cathepsin L, cathepsin S
	SerpinB4	Squamous cell carcinoma antigen 2 (SCCA2)	VVEL⇣SSPS	Cathepsin G, chymase
	SerpinB5	Maspin		Non-inhibitory
	SerpinB6	Placental thrombin inhibitor (PTI); cytoplasmic antiproteinase (CAP)	MMMR⇣CARF	Thrombin, plasmin, chymotrypsin, cathepsin G
	SerpinB7	Megsin	IVEK⇣QLPQ	
	SerpinB8	Cytoplasmic antiproteinase 2 (CAP2)	RNSR⇣CSRM	Furin, thrombin, subtilisin A
	SerpinB9	Cytoplasmic antiproteinase 3 (CAP3)	VVAE⇣CCME	Granzyme B, caspase 1, subtilisin A
	SerpinB10	Bomapin	IDIR⇣IRVP	Thrombin
	SerpinB11	Epipin	IAVK⇣SLPM	
	SerpinB12	Yukopin	VSER⇣SLRS	Trypsin, plasmin
	SerpinB13	Headpin	FTVT⇣SAPG	
Clade C	SerpinC1	Antithrombin	IAGR⇣SLNP	Thrombin; Factor Xa
Clade D	SerpinD1	Heparin cofactor II	FMPL⇣STQV	Thrombin
Clade E	SerpinE1	Plasminogen-activator inhibitor 1	VSAR⇣MAPE	Plasminogen activators (tPA; uPA)
	SerpinE2	Protease nexin-1	LIAR⇣SSPP	Plasminogen activators (tPA; uPA), thrombin
	SerpinE3	—	LLKR⇣SRIP	—
Clade F	SerpinF1	Pigment epithelial-derived factor		Non-inhibitory
	SerpinF2	Alpha-2 antiplasmin	AMSR⇣MSLS	Plasmin
Clade G	SerpinG1	C1-inhibitor	SVAR⇣TLLV	C1 proteinase
Clade H	SerpinH1	HSP-47		Non-inhibitory
Clade I	SerpinI1	Neuroserpin	AISR⇣MAVL	Plasmin, plasminogen activators (tPA; uPA)
	SerpinI2	Myoepithelium-derived serine proteinase inhibitor (MEPI); Pancpin	IPVI⇣MSLA	

For inhibitory serpins, the reactive centre loop (RCL) sequences are defined as P4–P4′, where (⇣) is the cleavage site for the target proteinase. Previously identified targets are also listed. List modified from [1,5,11] and RCL sequences confirmed using the UniProt Knowledgebase [46].

**Figure 1.**
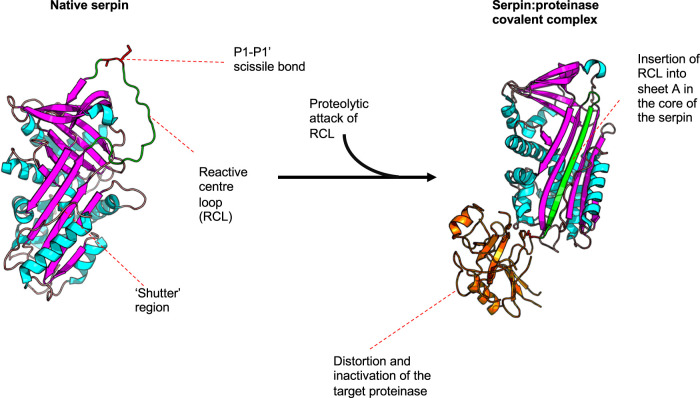
Serpin structure and inhibitory mechanism. Despite significant differences in sequence, the serpin superfamily display remarkable structural similarity. Serpins have 2–3 β-sheets surrounded by 7–9 α-helices and a reactive centre loop (RCL), which acts as a bait region for inhibitory serpins. Some conserved residues do exist amongst family members, for example, in the shutter region. Serpin inhibition begins with proteolytic attack of the RCL by a proteinase. Under normal circumstances, cleavage by a serine proteinase involves the formation of a covalent acyl-enzyme intermediate, followed by a deacylation step to release the cleaved products. In serpin inhibition, initial interaction with a proteinase is followed by the rapid and significant conformational of the serpin and distortion of the enzyme active site, such that deacylation cannot occur. This results in a hyperstable, covalent serpin:proteinase complex (right). Figure generated in PyMol, using structures imported from the Protein Data Bank — ID 1QLP [12], left; 1EZX [10], right.

Inhibitory serpins have an elegant mode of inhibition. Monomeric, serpins exist in a constrained (metastable, M-state) conformation until they interact with a proteinase. Often described as ‘molecular mousetraps’, the RCL of inhibitory serpins acts as a ‘bait region’ which contains a sequence of amino acids targeted by specific proteinase(s) [7]. Upon initiation of cleavage, the serpin undergoes a rapid switch, whereby the RCL is inserted into a β-sheet within the main body of the serpin [8–10]. As this occurs prior to the ‘deacylation’ step of proteolysis, the proteinase remains covalently attached to the serpin as an acyl-enzyme intermediate. This mode of suicide inhibition involves a huge conformational change which moves the proteinase ∼70 Å to the other side of the serpin protein [9] and renders a hyperstable serpin : proteinase complex (relaxed; R-state). The molecular dynamics of the serpin mechanism has both perplexed and fascinated the structural biology field for decades, and has been investigated and reviewed extensively elsewhere (see [11,12]). Interestingly, evolutionary analyses suggest that different serpins have emerged through gene duplication events, evolving by speciation to perform particular physiological roles, rather than specific inhibition of a proteinase [1,6]. The crystal structures in Figure 1 depict the important regions of the serpin and the structural changes which occur upon complex formation with a target proteinase.

### Cartilage destruction in osteoarthritis

Cartilage is a tissue which covers the end of long bones, lacks any vasculature or innervation, and has a single cell type — the chondrocyte. Composed of an organised extracellular matrix (ECM), it consists predominantly of type II collagen providing structural integrity, and the proteoglycan aggrecan, which provides compressive strength through osmotic water retention. Central to osteoarthritis (OA) is the destruction of this tissue and the exposure of the underlying bone, a process driven by metalloproteinases [13]. In particular, a disintegrin and metalloproteinase with thrombospondin motifs (ADAMTS) enzymes drive the initial loss of cartilage aggrecan, while matrix metalloproteinase (MMPs) can together degrade all the components of cartilage. Of these, in particular, it is the soluble collagenases, such as MMP-13, which are responsible for pathological collagen cleavage. Metalloproteinases are inhibited by a cognate family of inhibitors, the tissue inhibitor of metalloproteinases (TIMPs), of which there are four in humans (TIMP-1, -2, -3 -4) and provide a delicate balance between synthesis and degradation.

**Figure 2.**
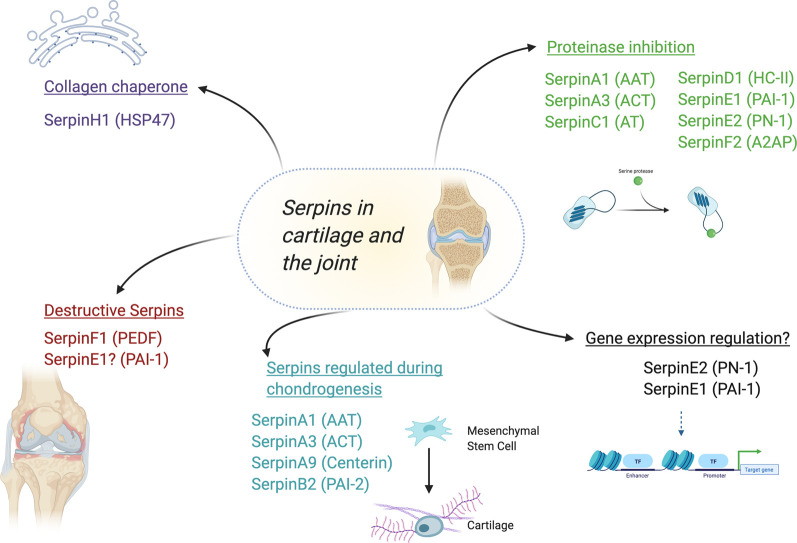
The putative roles and regulation of serpins in the joint. Abbreviated common names are shown in brackets for reference: SerpinA1 — AAT (alpha-1 antitrypsin); SerpinA9 (Centerin) — SerpinA3 — ACT (alpha-1 antichymotrypsin); SerpinB2 — PAI-2 (plasminogen Activator Inhibitor-2); SerpinC1 — AT (antithombin); SerpinD1 — HC-II (heparin cofactor II); SerpinE1 — PAI-1 (plasminogen activator inhibitor-1); SerpinE2 — PN-1 (protease nexin-1); SerpinF1 — PEDF (pigment epithelium-derived factor); SerpinF2 — A2AP (alpha-2 antiplasmin); SerpinH1 — HSP47 (heat shock protein 47).

More recently important roles for a different family of extracellular proteinases — the serine proteinases — has emerged in OA. Serine proteinases can activate proMMPs, cleave cellular receptors and cytokines as well as destroy the ECM directly [14–18]. The largest family of inhibitors of these proteinases are the serpins, which until now have not been interrogated collectively in this tissue. This review is not exhaustive, but will cover important studies relating to this unique superfamily of proteins in cartilage biology and OA. The currently identified roles for serpins in this context are summarised in Figure 2.

## Serpins in cartilage and osteoarthritis

### Clade A — the ‘antitrypsin-like’ serpins

This clade forms the largest extracellular serpin sub group; containing 11 genes and 2 pseudogenes [1]. Perhaps the most studied members of this clade are SerpinA1 (also known as alpha-1 antitrypsin or alpha-1 proteinase inhibitor) and SerpinA3 (alpha-1 antichymotrypsin). These serpins inhibit several proteinases but association kinetics are most favourable for the neutrophil serine proteinases (NSPs) — neutrophil elastase and proteinase-3 in the case of SerpinA1, and cathepsin G for SerpinA3 [19]. Both serpins are described as ‘acute phase’ proteins, which are synthesised upon initiation of systemic inflammation — predominantly by the liver — and function to down-regulate the inflammatory response [20]. Interestingly, both SerpinA1 and SerpinA3 are abundantly expressed by chondrocytes [21]. Perhaps underscoring the importance of SerpinA1 and SerpinA3 in cartilage biology, both are also markedly induced during chondrogenic differentiation from mesenchymal stem cells, and have been previously earmarked as putative differentiation markers [22]. In addition, SerpinA9 (centerin) is also induced when chondrogenesis is stimulated by kartogenin *ex vivo* [23]. It is not yet clear what if any role these serpins have in chondrogenesis and whether this is due to their inhibitory function. Indeed, both SerpinA1 and SerpinA3 have important functions outside of their inhibitory activity which could be important within the context of cartilage biology [24,25].

As early phase proteins, it is perhaps unsurprising that levels of SerpinA1 and SerpinA3 have been shown to be increased in the serum and synovial fluid of rheumatoid arthritis patients [26] and SerpinA1 expression is increased in response to inflammatory cytokines in chondrocytes [27]. However, the expression of this serpin appears to be reduced in OA cartilage, and lower levels have been observed in OA synovial fluid compared with non-OA controls [22,28]. Intraperitoneal injection of SerpinA1 protects against destruction in the collagen-induced arthritis model of inflammatory arthritis [29], and this serpin can block collagen release from cytokine-stimulated bovine cartilage *ex vivo* [30]. The localisation of both SerpinA1 and SerpinA3 within the cartilage may also provide insights as to their function in the tissue. A cross-sectional quantitative proteomic study of cartilage observed that both serpins were located predominantly in the superficial cartilage layer [31]. This perhaps reflects a well-positioned defence against damaging neutrophil proteinases, which through knock-out studies have been shown to be essential for the progression of collagen-induced arthritis, a model of rheumatoid arthritis [32]. Emerging evidence suggests proteinases such as neutrophil elastase may also have a role in OA [33–35] meaning changes in levels of these serpins might be of particular importance in this context.

### Clade B — the intracellular serpins

Even amongst the serpin superfamily, the clade B serpins are unique. Lacking a signal peptide, they form an intracellular clade, which likely predates extracellular serpins [1]. Despite this, several studies have reported clade members extracellularly, suggesting other modes of secretion into the extracellular space, beyond the classical secretory pathway [36]. They show remarkable similarity in gene structure across the clade (8 exons, 7 introns) and are present in mammals and birds, but not in earlier model organisms such as *Caenorhabditis elegans* or *Drosophila melanogaster*, leading to suggestions the clade is at least 300 million years old [36]. Members of this clade have diverse roles including regulating cell growth, apoptosis, immunity and protecting cells from intracellular proteolysis [37]. Few studies have investigated B clade serpins in cartilage, although it has been shown that SerpinB2 (plasminogen activator inhibitor (PAI)-2), like SerpinA9, is markedly induced during cartilage formation following MSC treatment with kartogenin [23]. Interestingly, proteomic analyses of urine suggest that SerpinB1 (leukocyte elastase inhibitor; LEI) and SerpinB3 were both significantly down-regulated in older patients with OA compared with urine from younger healthy controls without OA [38].

### Clade C — antithrombin

This clade has just one member, SerpinC1 (commonly known as antithrombin), which acts a major inhibitor of coagulation enzymes and has a circulating blood concentration of 0.15 g/l [39]. SerpinC1 is poorly expressed by chondrocytes [21], but this serpin is detectable in synovial fluid with increased SerpinC1 : proteinase complexes observed in both the OA and RA patients [40]. Interestingly, SerpinC1 requires heparin for maximal inhibitory activity, which is commonly administered as an antithrombotic. This glycosaminoglycan (GAG) increases the association constant approximately 300-fold, resulting from a shift in the main sheets within the serpin core, and an extrusion of the RCL, making it more exposed to proteolytic attack [41]. The potential significance of cartilage-derived GAGs in regulating serpin activity is explored further in section ‘ECM binding’.

### Clade D — heparin cofactor II

This clade also only contains one member — SerpinD1 (also known as Heparin Cofactor II) which is rapid inhibitor of thrombin, but like SerpinC1, only in the presence of GAGs; including heparin, heparan sulfate or dermatan sulfate. Despite sharing 30% sequence identity with SerpinC1, little is known about its physiological function, although a role in the response to vascular injury is likely [42,43]. SerpinD1 has been shown to bind strongly to the small leucine-rich proteoglycans decorin and bigylcan [44], both of which are abundant in cartilage. Proteomic analysis of synovial fluid has demonstrated that SerpinD1 is elevated in OA compared with healthy controls [45].

### Clade E — plasminogen activator inhibitor-1 and proteinase nexin-1

This clade is home to three serpins, only two of which are well described — SerpinE1 (plasminogen activator inhibitor-1) and SerpinE2 (protease nexin-1). These serpins share 40% sequence identity and significant structural overlap [46]. Like many serpins, they target trypsin-like serine proteinases due to an arginine residue in the P1 position in their RCL [5] but they also share an additional commonality in other RCL amino acids (Table 1). SerpinE1 is a well-established regulator of fibrinolysis through inhibition of urokinase and tissue-type plasminogen activators (uPA and tPA, respectively; [47]). SerpinE2 is also a potent inhibitor of uPA, as well as the coagulation proteinase, thrombin [48]. It is important to recognise that despite the biochemical similarities, the physiological functions of these serpins display marked differences, perhaps best evidenced by phenotypes of mice deficient for these serpins. *SerpinE1*^−/−^ mice display altered clot lysis [49], whilst *SerpinE2*^−/−^ mice display neurological abnormalities and males are infertile [50,51]. As with the clade A serpins, both *SerpinE1* and *SerpinE2* are induced by pro-inflammatory cytokines in chondrocytes [52–54] and *SerpinE1* has been shown to be induced by both mechanical loading and fluid shear stress [55,56]. In OA, some studies have observed decreased levels of both *SerpinE1* [57] and *SerpinE2* [21] in the cartilage, perhaps suggesting an increased proteolytic load. Indeed, we have demonstrated that both serpins are able to protect against cartilage collagen breakdown in an *ex vivo* model of cartilage destruction, likely through the inhibition of proteolytic activators of MMPs (as yet unpublished observations). Intra-articular SerpinE2 administration was also able to protect against joint destruction in a rabbit model [58]. Santoro and colleagues demonstrated that SerpinE2 can block interleukin (IL)-1 induced expression of MMP-13 in chondrocytes, in a mechanism which appears to involve ERK, NFκB and AP-1 [53] with similar observations demonstrated in the cartilage of the intervertebral disc [59]. Neither study investigates the involvement of serine proteinases in these observations, and it is possible that SerpinE2 may have chondroprotective roles outside of proteinase inhibition. Interestingly, an inverse observation has been made for SerpinE1, as murine chondrocytes from *SerpinE1*^−/−^ mice show a reduction in IL-1 induced *Mmp13* expression [60]. These mice also display accelerated subchondral osteopenia compared with wild-type mice following surgical induction of OA in ovariectomized female mice [61].

The effect of clade E serpin genes on joint pathology is not exclusively at the protein level. In 2019, Shen and colleagues identified a circular RNA (*circSERPINE2*) which is down-regulated in OA and appears to regulate catabolic factors in the cartilage, acting as a sponge for microRNAs. The authors demonstrated that adenoviral overexpression of *circSERPINE2* was able to protect cartilage in a rabbit OA model [62].

### Clade F — pigment derived epithelial factor and alpha-2 antiplasmin

This clade consists of 2 members SerpinF1 and SerpinF2. SerpinF1 is perhaps better known as pigment epithelium-derived factor (PEDF). Devoid of inhibitory activity, this serpin has anti-angiogenic, neurotrophic and differentiation-inducing properties, and numerous studies have described its role cancer [63]. SERPINF1 acts predominantly through the cognate receptor PEDFR, and its action appears to be highly regulated by binding to ECM components [64–66]. In the joint, the role of SerpinF1 is catabolic, promoting cartilage destruction. Indeed, SerpinF1 has been reported to be increased in OA cartilage [45], and a recent study demonstrated that *SerpinF1* deficiency reduces cartilage damage in an age-dependent manner in the murine monoiodoacetamide OA model, and overexpression in chondrocytes enhances cytokine-induced expression of catabolic MMPs [67]. In support of these observations, a different study demonstrated that stimulation of chondrocytes with recombinant SERPINF1 led to a catabolic phenotype and chondrocyte terminal differentiation [68].

The other serpin in this clade, SerpinF2 (also known as alpha-2 antiplasmin) is the primary inhibitor of plasmin, a proteinase which functions to cleave fibrin to promote clot disruption. Plasmin can also activate MMPs and contribute to ECM turnover [14]. In haemophilic mice, it has been demonstrated than fibrinolytic proteinases are liberated from the synovium during hemoarthrosis [69] and that intra-articular SerpinF2 administration was able to reduce cartilage damage and synovitis [70]. Any protective effect of this SerpinF2 in the OA joint has not yet been investigated, however.

### Clade H — HSP47 — an essential collagen chaperone

The only serpin in its clade, SerpinH1 (also known as heat shock protein (Hsp)47) is a non-inhibitory serpin which has a primary function as a collagen chaperone within the endoplasmic reticulum (ER). Unlike other chaperones, this serpin appears to have only one client protein, and binds at regular sites along the collagen triple helix [71]. Cells deficient for this protein show accumulation of collagen aggregates in the ER [72]. It is, therefore, essential for new collagen synthesis and normal cartilage development and homeostasis. Indeed, homozygous missense mutations in the *SERPINH1* gene result in *osteogenesis imperfecta* [73] and mice deficient for *SerpinH1* die shortly after birth, exhibiting generalised chondrodysplasia and bone abnormalities [74]. Phylogenetic analysis demonstrates the *SERPINH1*/*HSP47* gene to be present in cartilaginous fish such as the Japanese lamprey, dating back approximately 500 million years [75].

A role for SerpinH1 in OA is not yet clear. Changes in levels of collagen synthesis during different stages of cartilage catabolism in OA are, however, well described [76]. The corollary of which is that an essential collagen chaperone will also have an important role in such changes, but this has not yet been determined experimentally.

## Common modes of serpin regulation and their implications for joint tissues

**Figure 3.**
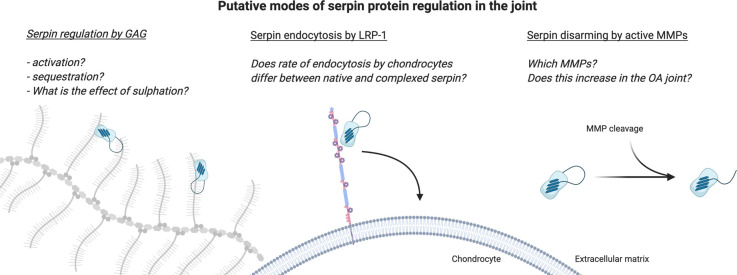
Modes of serpin protein regulation with direct relevance to joint tissues and osteoarthritis. (**A**) At the protein level, serpins are well described GAG-binding proteins, the interaction with which can significantly alter their inhibitory capacity. Cartilage is rich in sulfated GAG in proteoglycans such as aggrecan. (**B**) Endocytosis is emerging as a major regulator of the levels of proteinases and their inhibitors in cartilage. ADAMTS-4, ADAMTS-5, MMP-13 and TIMP-3 are all endocytosed by LRP-1 in chondrocytes, and increased shedding of the LRP-1 receptor in OA has likely importance for the regulation of cartilage matrix proteolysis. Several serpins are well-described ligands for LRP-1 in other tissues. (**C**) Specific inactivation of serpins by MMPs occurs in close proximity to the canonical serine proteinase cleavage site. This results in disarming of the serpin by removal of the RCL. Active MMPs in the OA joint likely contribute to the proteolytic burden by significant inactivation of serpins.

Serpin proteins display exceptional characteristics both structurally and evolutionary. Perhaps as a result, many members also share common modes of regulation as summarised in Figure 3.

### ECM binding

Cartilage ECM is rich in proteoglycan; predominantly aggrecan, but also more minor components such as biglycan, decorin and perlecan. GAG chains provide the correct osmotic potential in the cartilage to retain water and resist compression, but they also act as a major regulators of cartilage ECM proteins, including both metalloproteinases and their inhibitors [77,78]. GAGs can not only bind and sequester serpins, but for some also enhance their inhibitory activity, by causing large changes in conformation, making proteolytic attack of the RCL more favourable (see [79]). Many serpins are recognised as binding to GAGs and protein structures are often crystallised in complex with them (usually heparin; [48,80]). It is plausible that serpin inhibitory activity can be enhanced in the presence of GAGs within cartilage, and as our understanding of the role of serpins in this tissue improves, so too will their association with, and the relevance of, particular cartilage GAG chains. Sulphation patterns of GAGs are critical to the binding of serpin exosites [81]. In cartilage, sulphation has been shown to be important for GAG binding of ADAMTS-5 and TIMP-3 [78], and the sulphation pattern of important cartilage GAGs changes in OA, which may impact on their function in the tissue [82,83].

### Endocytosis

Endocytosis is becoming established as a mode of metalloproteinase regulation in cartilage and OA. The endocytic receptor LRP-1 is a large transmembrane protein which plays a critical role in the regulation of MMPs, ADAMTS proteinases and TIMPs by binding and removing them from the extracellular space [84–87]). Indeed, there is evidence that LRP-1 and GAGs compete for the binding of TIMP-3 [78]. Serpin binding to LRP-1 is well established; initial observations suggested the involvement of a ‘serpin/enzyme complex (SEC) receptor’ which was later identified to be LRP-1 [88]. Many serpin : proteinase complexes have been identified as LRP-1 ligands (see [88,89]). It has been suggested that for most serpins, little LRP-1 binding of monomeric serpin (native or cleaved) occurs, whereas those in complex with a proteinase are readily endocytosed [88], implying a common mode of clearing complexes from the extracellular milieu. LRP-1 shedding is increased in OA cartilage [90], but it is not yet clear how this affects serpin levels within the tissue.

### Proteolytic inactivation by MMPs

Interaction between serine and metalloproteinase families is well established as an important factor in the breakdown of cartilage in arthritis [14], and serpin inactivation by MMPs is another example. Cleavage occurs within the serpin RCL, removing the bait region and rendering the serpin inactive. *In vitro*, SerpinA1 and SerpinA3 are inactivated by MMP1, MMP-2 and MMP-3 [91,92], while SerpinA1 has also been demonstrated to be inactivated by MMP-9 *in vivo* [93]. Furthermore, SerpinF2 is rapidly inactivated by MMP-3 [94] and we have recently demonstrated that MMP-13 — the major OA collagenase — is also able to rapidly inactivate SerpinA1 (as yet unpublished observations). It is not yet clear what purpose this regulation serves, but it is likely to have a significant impact on the proteolytic burden in tissues where serine and metalloproteinase activity is of particular importance, such as the OA joint. Indeed, proteolytic inactivation of serpins in synovial fluid from arthritis patients has been observed [95,96] but it is unclear the degree to which serpin ‘disarming’ exacerbates catabolism. The development of neo-epitope antibodies specific for MMP-cleaved serpins could provide the foundation for understanding how serpins control the proteolytic environment and how this changes with the progression of joint destruction in OA.

## Discussion: opportunities, challenges and future directions

Our understanding of the role of serine proteinases in cartilage biology and OA has accelerated in recent years, which prompts re-appraisal of their largest family of endogenous inhibitors. Serpins as a protein family have been largely overlooked in joint health and disease due — at least in part — to confusing nomenclature. For example, inhibitory serpins were often given names based on a target which is unlikely to be their major target *in vivo*. The likely physiological proteinase target of alpha-1 antitrypsin (SerpinA1) is not trypsin, but rather neutrophil elastase for which its kinetics are overwhelmingly favourable (*K*_a_ = 6.5 × 10^7 ^M^−1 ^s^−1^; [97]). Recent standardised nomenclature and separation into clades based on sequence similarities have provided some clarity, but multiple names has led to many observed changes in serpin transcript or protein levels — identified through ‘omic technology’ — being largely ignored when ascribing key pathways or proteins in disease mechanisms [98,99]. Here for the first time, the superfamily is discussed as a whole, in the context of their relationship with cartilage and OA.

In OA, several studies have demonstrated inhibitory serpins which are markedly down-regulated [21,22,99], perhaps implying decreased proteolytic inhibition, the potential consequences of which could be uncontrolled proteolysis. This appears paradoxical, as several serpins (including SerpinA1 [27], SerpinA3 [100], SerpinE1 [101] and SerpinE2 [53]) can be induced by pro-inflammatory cytokines which may also play a role in driving OA [102,103]. However, it is important to recognise that periodic expression changes do not always mirror long-term observations in a slow degenerative disease. Furthermore, in an environment where MMPs can inactivate some serpin family members, total serpin protein is likely an over-representation of functional serpin levels in the joint. Some serpins are also inactivated by oxidative stress, which is considered a principal driver of OA [104,105]. SerpinA1, SerpinC1, SerpinE1, SerpinF2 and SerpinG1 are all known to be inactivated by oxidation [106,107]. In the RA joint, SerpinA1 inhibitory activity is depressed [108] and oxidation-associated inactivation has been observed following exercise [109]. Further work is required to explore the degree to which serpins are inactivated by proteolysis and oxidation in the OA joint, but both are likely to have a role in reducing functional inhibitor levels.

It is likely that changes in the levels of inhibitory serpins could promote cartilage breakdown, through an increased activity of serine proteinases, which are themselves important in destruction. Proteinases which are known proMMP activators (reviewed in [14]), and are well established to be controlled by serpin inhibition include neutrophil elastase, plasmin and proprotein convertases such as furin (see Table 1), although there are likely to be others. Which serpins are essential for chondroprotection through antiproteinase activity, and the stage of OA in which they are most important, are yet to be elucidated, however. Using transgenic mice deficient for particular serpins in animal models of OA will help in this endeavour. It should be noted, that some family members display differences in the number of paralogues between species which will be a significant hurdle in this respect. For example, whilst humans have one *SERPINA1* gene, mice have six members (*Serpina1a-f*), and although humans have one *SERPINA3* gene, the mouse has nine paralogues (*Serpina3a-c, f-n*). Functional comparisons are limited, but a recent study used CRISPR/Cas9 technology to remove 5 *Serpina1* paralogues and demonstrated that mice exhibited lung emphysema [110], reflecting patient phenotype of the human genetic condition alpha-1 antitrypsin deficiency (AATD), a disorder with significant lung dysfunction due to uncontrolled neutrophil elastase activity. In the future, the use of such mice could inform studies investigating the effect of *Serpina1* deficiency in murine experimental arthritis. Fortunately, for most serpins, one human gene is mirrored with a single orthologue in rodents, and indeed transgenic animals have begun to be used successfully to understand the importance of these serpin genes in diseases, including OA [61,67,74].

Serpins themselves are already licenced for treatments in numerous disorders [5]. Perhaps the most established is augmentation therapy for AATD, with weekly IV infusion of SerpinA1 protein derived from pooled human plasma [111,112]. Another example is the prophylactic treatment of hereditary angioedema with SerpinG1 (C1 esterase inhibitor; [113]). Should serpin augmentation for arthritic diseases be beneficial, systemic administration would likely deliver little to the joint capsule — presenting a pharmacokinetic challenge — although intra-articular injection could be a viable alternative. The unique serpin mechanism lends itself to the production of recombinant ‘designer serpins’ which may result in altered target specificity or improved stability [5]. For example, SerpinE1 is a uniquely unstable protein, with a half-life of ∼1–2 h. Four-point mutations can be made which remarkably increase this time by over 100 h, without significantly compromising serine proteinase inhibition, making this mutant more suitable for many research applications [114,115]. It seems likely that the recombinant production of serpins with improved properties may well influence how these proteins are used in the future both in a research environment and perhaps also therapeutically.

There is a strong precedent for the successful inhibition of serine proteinases and indeed the use of serpins themselves to treat a multitude of diseases. With recent evidence solidifying the importance of this protein superfamily in OA, understanding serpin biology, physiological targets and regulation in these tissues warrants further investigation and has the potential to offer new insights and pathways to treatment.

## Perspectives

- *Highlight the importance of the field*: Cartilage destruction is central to osteoarthritis, where an imbalance of proteinases to their inhibitors promotes catabolism. The largest family of serine proteinase inhibitors are the serpins, an ancient and remarkable superfamily of proteins, which, despite their likely role in controlling proteolytic pathways, are often ignored in the context of joint disease.
- *A summary of the current thinking*: Serpins control the proteolytic activity of serine proteinases and play a crucial role in normal joint homeostasis. Non-inhibitory activity of serpins also has important roles in cartilage function, for example in collagen chaperoning.
- *A comment on future directions*: Serpins represent important mediators for controlling the proteolytic environment. A better understanding of their specific roles in joint biology will be crucial if this potential is to be harnessed for therapeutic gain in OA; either using serpins themselves, or as tools to identify particular proteinases which may be of interest for drug discovery.

## Open Access

Open access for this article was enabled by the participation of University of Liverpool in an all-inclusive *Read & Publish* pilot with Portland Press and the Biochemical Society under a transformative agreement with JISC.

## Abbreviations

ADAMTS: a disintegrin and metalloproteinase with thrombospondin motifs
ATD: alpha-1 antitrypsin deficiency
ECM: extracellular matrix
GAG: glycosaminoglycan
HSP: heat shock protein
IL: interleukin
LRP-1: low-density lipoprotein receptor-related protein 1
MMP: matrix metalloproteinase
NSP: neutrophil serine proteinases
OA: osteoarthritis
RCL: reactive centre loop
Serpin: serine proteinase inhibitor
TIMP: tissue inhibitor of metalloproteinase
tPA: tissue plasminogen activator
uPA: urokinase plasminogen activator
